# Outcomes for Medicaid Patients with Colorectal Cancer Are Improved in Affluent Neighborhoods, but Disparities Persist

**DOI:** 10.3390/cancers17091399

**Published:** 2025-04-22

**Authors:** Kaelyn C. Cummins, Mohamad El Moheb, Chengli Shen, Susan J. Kim, Russell Witt, Samantha M. Ruff, Allan Tsung

**Affiliations:** Department of Surgery, University of Virginia, Charlottesville, VA 22903, USA; kcc7fa@uvahealth.org (K.C.C.); krb3ym@uvahealth.org (M.E.M.); gve6nm@virginia.edu (C.S.); pxa3kp@uvahealth.org (S.J.K.); syd3xz@uvahealth.org (R.W.); jzp9rg@uvahealth.org (S.M.R.)

**Keywords:** colorectal cancer, Medicaid, socioeconomic status, outcomes, National Cancer Database, income, education, survival

## Abstract

Background: Socioeconomic status (SES) significantly influences outcomes in colorectal cancer (CRC) patients, with those from low-SES backgrounds facing worse prognoses. However, living in an affluent neighborhood may mitigate some of these disparities through environmental advantages. This study investigates whether Medicaid-insured CRC patients, as a proxy for low individual SES, experience better outcomes when residing in high-SES neighborhoods. Methods: Using the National Cancer Database, we examined Medicaid CRC patients, stratifying them by neighborhood SES indicators: median household income and education level. Patients in the highest and lowest quartiles of income and education were compared. Medicaid patients from the highest-SES neighborhoods were compared to the general population. Multivariable regression models analyzed 30- and 90-day postoperative mortality, overall survival (OS), and time from diagnosis to treatment initiation and surgery. Results: CRC patients in high-income neighborhoods began treatment earlier (coefficient −1.847, *p* = 0.015) and exhibited improved OS (HR 0.810, *p* < 0.001) compared to those in low-income neighborhoods, irrespective of education level. Similarly, patients in high-education neighborhoods started treatment sooner (coefficient −3.926, *p* < 0.001) and had better OS (HR 0.897, *p* < 0.001). No differences were observed in time to surgery or postoperative mortality. Despite these advantages, Medicaid patients in high-income (HR 1.130, *p* < 0.001) and high-education (HR 1.209, *p* = 0.002) areas still had worse OS compared to non-Medicaid patients. Conclusions: Higher neighborhood SES is associated with a significant survival benefit for Medicaid CRC patients, but these patients still lag behind their non-Medicaid counterparts. Understanding the mechanisms by which neighborhood SES influences cancer outcomes could inform targeted interventions to close the survival gap.

## Simple Summary

Outcomes in colorectal cancer patients are significantly influenced by patients’ socioeconomic status, but interplay between individual and neighborhood socioeconomic status is not well characterized. The goal of this retrospective study was to shed light on this interplay by investigating the effect of neighborhood socioeconomic status in colorectal cancer patients with low individual socioeconomic status. Patients living in affluent neighborhoods had faster treatment initiation and better overall survival than those living in low-socioeconomic status areas, but their outcomes remained worse than the general population. These findings can be used to inform future study, develop targeted interventions, and help close the survival gap related to socioeconomic inequality in these vulnerable patients.

## 1. Introduction

Colorectal cancer (CRC) is the third most commonly diagnosed cancer and cause of cancer-related death in the United States. In the United States alone, over 150,000 patients are newly diagnosed with CRC, and an estimated 50,000 patients will die of CRC annually. Notably, the disease is increasingly being diagnosed at later stages and in younger patients, making a thorough understanding of CRC, its risk factors, and effective treatments more important than ever [1]. The global burden is even more significant, with an estimated 1.9 million new cases and nearly 1 million deaths in 2020 [2].

CRC is frequently diagnosed via screening [3] and then treated with a combination of surgery, chemotherapy, immunotherapy, and/or radiation, depending on cancer stage and location. After treatment, surveillance for recurrent disease is recommended [4,5]. At each of these steps, more disadvantaged patients with less access to health care may fall through the cracks.

Socioeconomic status (SES) plays a significant role at all levels of health care. However, it is important to note that SES is not a singular, quantifiable value, but rather an umbrella term that encompasses various factors that impact patients throughout the healthcare process. Understanding these nuances creates an opportunity to propose targeted interventions and improve patient outcomes.

Previous studies have shown that all-cause [6], lung cancer [7], breast cancer [8], and indeterminate cancer [9] mortality were worse in lower-income neighborhoods, even when controlling for individual patients’ income. In CRC specifically, lower SES has correlated with increased incidence [10,11,12,13], later disease stage at presentation [14,15,16,17], and worse overall outcomes [16,17]. A recent study concluded that neighborhood SES was the greatest contributing factor to the disparity in overall survival between White and Black patients, with 29% of the difference in this outcome attributable to neighborhood SES [18]. Although much of this research has been done in American cohorts, the negative influence of low SES on CRC outcomes holds in countries outside of the United States [19,20,21,22,23,24,25,26] and on a global scale [27]. This surfeit of evidence suggests that patients’ environments greatly impact their health outcomes.

While CRC mortality is worse in low-SES neighborhoods [16,17,28,29,30,31,32,33], this relationship between individual and neighborhood SES has not been well studied. More research is needed to fully characterize the inequalities faced by low-SES patients with CRC in order to design and implement actionable change. The underlying mechanism of how environment impacts CRC disparities is still unclear. Furthermore, the literature has yet to elucidate how environment and individual SES influence each other in patients with CRC. In this study, we sought to control the influence of individual SES in order to specifically evaluate the impact of patients’ surroundings on their CRC outcomes. To do this, we focused on patients with individual low SES from different socioeconomic environments.

## 2. Materials and Methods

This study was deemed exempt from informed consent by the University of Virginia Institutional Review Board due to the de-identified nature and public availability of the dataset.

### 2.1. Study Population

De-identified data were sourced from the National Cancer Database (NCDB) using the 2020 participant user file. The NCDB is a joint project of the Commission on Cancer (CoC) of the American College of Surgeons and the American Cancer Society and covers over 70% of cancer diagnoses in the United States [34]. The CoC’s NCDB and the hospitals participating in the CoC’s NCDB are the source of the de-identified data used herein; they have not verified and are not responsible for the statistical validity of the data analysis or the conclusions derived by the authors.

Medicaid is the largest health insurance program in the United States and insures nearly one-quarter of Americans. Its eligibility is largely based on income, with a majority of states offering coverage to adults with incomes 138% or less than the federal poverty level [35]. Medicaid insurance status therefore served as a surrogate for individual SES, which has been used previously as a surrogate for low-income status [36]. Patients ≥ 18 years old in the NCDB covered by Medicaid insurance who were diagnosed with CRC of any stage and any histology between the years 2004 and 2020 were included. There were no systematic exclusion criteria.

For comparison with non-Medicaid patients, all patients ≥ 18 years old in the NCDB not enrolled in Medicaid who were diagnosed with CRC of any stage and any histology between the years 2004 and 2020 were included. This included patients whose insurance status was unknown.

### 2.2. Study Groups and Outcomes

The initial analysis involved two distinct comparisons to assess the impact of environmental SES factors on cancer care: one focused on income, and the other on education. The study population was first stratified into quartiles based on the median household income associated with the patient’s zip code, using data from the time period corresponding to the patient’s diagnosis. To emphasize the contrast in socioeconomic conditions, patients within the median two quartiles were excluded, and patients from the highest-income quartile were compared against those from the lowest quartile. A similar stratification was applied for educational attainment, using the proportion of residents with a high school diploma within each zip code. This was a comparison between patients from zip codes in the highest quartile of high school graduation rate against those in the lowest quartile.

Subsequently, a combined income/education analysis was conducted. Medicaid CRC patients from zip codes that were simultaneously in the highest or lowest quartile of income (HI vs. LI) and the highest or lowest quartile of education (HE vs. LE) were included. This generated four groups: HI/HE, HI/LE, LI/HE, and LI/HI. Outcomes of patients in these groups were then compared against one another.

Finally, overall survival in Medicaid patients with CRC living in the highest income and education quartiles was compared to overall survival in non-Medicaid patients.

The primary outcomes of interest were overall survival and mortality rates at 30 and 90 days post-surgery. Secondary outcomes were time from CRC diagnosis to the initiation of treatment and the time from diagnosis to surgical intervention. Short-term postoperative mortality was evaluated to specifically gauge the impact of surgical disparities, whereas other variables were selected to study overall cancer care.

### 2.3. Statistical Analysis

Univariable analysis was used to compare the baseline characteristics and outcomes of Medicaid patients with CRC patients living in areas of diverging SES as measured by median household income and educational attainment. Continuous variables were summarized as means with standard deviations and were compared using independent *t*-test, and categorical variables were summarized as frequencies with percentage and compared using Chi-Square test.

Multivariable linear regression models were utilized to evaluate differences in the time to initiation of treatment and time to surgery across study groups, adjusting for age, sex, race, ethnicity, comorbidities as quantified by Charlson–Deyo score [37], disease stage, and type of facility. The Charlson–Deyo score incorporates patients’ co-morbidities to predict long-term survival and is calculated in the NCDB. Furthermore, multivariable logistic regression models were developed to assess 30-day and 90-day postoperative mortality in the subset of patients who underwent surgery, adjusting for the aforementioned variables and any medical treatments administered (including chemotherapy and immunotherapy). Cox regression analysis was subsequently employed to analyze overall survival, adjusting for the previously mentioned factors and the surgical status of the patients. Patients with missing data were excluded from multivariable regression analyses. Multicollinearity was evaluated, with variance inflation factors all below 5. A sensitivity analysis was conducted to investigate the potential interaction between income and education levels. Statistical significance was determined by two-sided p-values of less than 0.05. For continuous variables, normality was assumed per the central limit theorem.

All statistical analyses were performed using Stata version 15 (StataCorp LLC, College Station, TX, USA).

## 3. Results

A total of 63,777 Medicaid patients across all zip codes were diagnosed with CRC between 2004 and 2020 and therefore qualified for analysis.

### 3.1. Income Analysis

**Figure 1:**
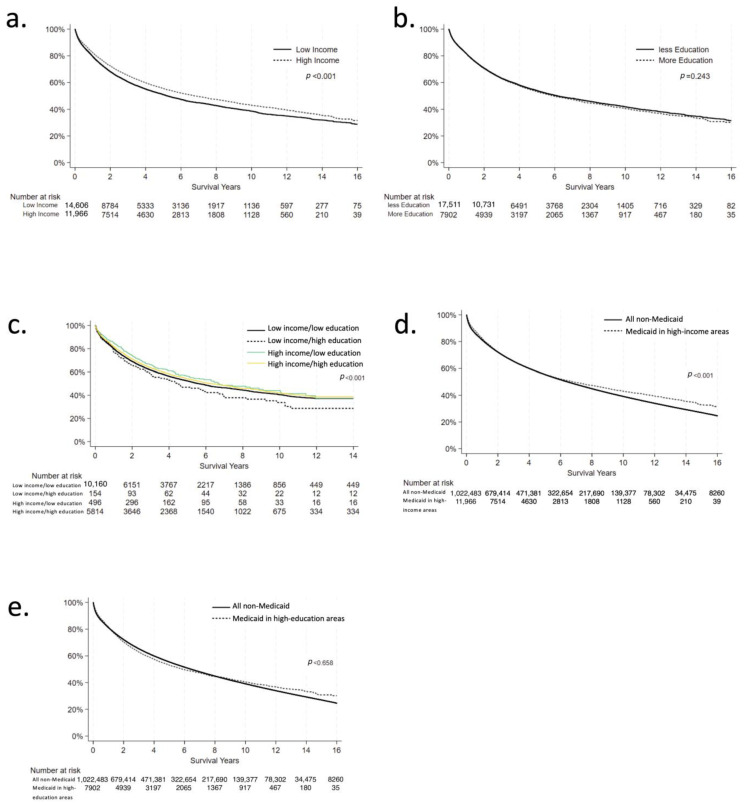
Unadjusted Kaplan–Meier curves for overall survival for NCDB patients with colorectal cancer diagnoses between 2004 and 2020. All curves truncated when n < 10 patients in any group. (**a**) Overall survival of Medicaid patients with colorectal cancer from low-income (solid line) and high-income (dotted line) zip codes. (**b**) Overall survival of Medicaid patients with colorectal cancer from low-education (solid line) and high-education (dotted line) zip codes. (**c**) Overall survival of Medicaid patients with colorectal cancer from low-income/low-education (black line), low-income/high-education (dotted line), high-income/low-education (green line), and high-income/high-education (yellow line) zip codes. (**d**) Overall survival of non-Medicaid patients with colorectal cancer (black line) compared to Medicaid patients living in the highest-income (dotted line) zip codes. (**e**) Overall survival of non-Medicaid patients with colorectal cancer (black line) compared to Medicaid patients living in the highest-income (dotted line) zip codes.

A total of 15,730 patients lived in the lowest-income quartile, and 13,046 lived in the highest-income quartile area (Table 1). Medicaid patients living in the highest-income quartile were more likely to be older (57.7 years vs. 55.6 years), White (68.1% vs. 51.2%) or Asian (11.6% vs. 2.7%), non-Hispanic (87.4% vs. 83.7%), have a lower Charlson–Deyo score (0.363 vs. 0.454), and be diagnosed with CRC at a lower stage (26.9% vs. 24.9% stage 0 or 1) compared to those living in lowest-income areas. When controlling for demographic factors, disease stage, treatment facility type, and comorbidities, patients in high-income neighborhoods initiated treatment significantly earlier than those in low-income neighborhoods (coefficient −1.847, *p* = 0.015; Table 2). There was no difference in time from diagnosis to surgery (coefficient −1.390, *p* = 0.299). The adjusted 30-day postoperative mortality was similar in patients living in high-income areas vs. those living in low-income areas (OR 0.793, *p* = 0.163), as was the 90-day mortality (OR 0.863, *p* = 0.211). Improved overall survival was associated with patients from high-income zip codes, both when unadjusted and when controlled for demographic factors (adjusted HR 0.810, *p* < 0.001; Figure 1a). Factors associated with worse overall survival included non-Hispanic ethnicity (HR 1.435, *p* < 0.001), White race (HR 1.063, *p* = 0.037 compared to Black patients; HR 1.305, *p* < 0.001 compared to Asian patients), older age (HR 1.014, *p* < 0.001), male sex (HR 1.133, *p* < 0.001), and higher disease stage (HR 8.017, *p* < 0.001 for stage 4 vs. stage 0).

### 3.2. Education Analysis

A total of 19,073 patients lived in the lowest-education quartile, and 8,375 lived in the highest-education quartile areas (Table 3). Once again, those living in the highest-education quartile areas were more likely to be older (57.5 years vs. 55.6 years), White (73.7% vs. 55.5%) or Asian (8.6% vs. 6.7%), non-Hispanic (93.1% vs. 74.0%), have a lower Charlson–Deyo score (0.367 vs. 0.761), and be diagnosed with CRC at a lower stage (25.9% vs. 24.8% stage 0 or 1) than those in the lowest-education quartile areas. Patients in the highest-education zip codes had a significantly shorter time from CRC diagnosis to treatment initiation (coefficient −3.926, *p* < 0.001), but there was no difference in time from diagnosis to surgery (coefficient −2.500, *p* = 0.097; Table 4). There was no difference in 30-day postoperative mortality (OR 1.047, *p* = 0.799) or 90-day mortality (OR 0.950, *p* = 0.694). There was no difference in overall survival when unadjusted; however, living in a high-education area was significantly associated with improved overall survival when adjusted for demographic characteristics (HR 0.897, *p* < 0.001; Figure 1b). Similar to the prior analysis, factors associated with decreased overall survival included older age (HR 1.011, *p* < 0.001), male sex (HR 1.133, *p* < 0.001), White race (HR 1.468, *p* < 0.001 compared to Asian patients), non-Hispanic ethnicity (HR 1.541, *p* < 0.001), and higher disease stage (HR 8.173, *p* < 0.001 for stage 4 vs. stage 0).

### 3.3. Combined Variable Analysis

A total of 16,558 patients met criteria for the combined analysis (Table 5). Of these, 10,817 patients lived in zip codes that were lowest-quartile in both income and education (LI/LE); 4345 lived in zip codes that were highest-quartile in both income and education (HI/HE); 79 lived in zip codes that were lowest-quartile in income and highest-quartile in education (LI/HE); and 1317 lived in zip codes that were highest-quartile in income and lowest-quartile in education (HI/LE). Consistent with the prior analyses, the HI/HE group initiated treatment earlier than the LI/LE after adjustment (coefficient −4.419, *p* < 0.001); there were no other differences in treatment initiation between groups. Additionally, there was no difference between any of the groups in timing to surgery or postoperative mortality. However, the LI/LE group had worse overall survival than both the HI/HE group (HR 0.827, *p* < 0.001) and the HI/LE group (HR 0.800, *p* < 0.022); this difference did not carry over when comparing LI/LE with LI/HE (HR 0.958, *p* = 0.768; Figure 1c).

### 3.4. Medicaid vs. Non-Medicaid Analysis

Given the positive association between survival and living in a high-SES environment, we sought to evaluate whether outcomes in low-SES individuals living in high-SES areas were comparable to the level of the general population, or whether disparities based on individual SES persisted. We compared overall survival between Medicaid patients living in the highest-income zip codes and the general population (Table 6), represented by the total population of non-Medicaid CRC patients (n = 1,117,466). We opted to include all non-Medicaid CRC patients rather than excluding uninsured patients to more closely approximate the general US population. When adjusted for demographic factors, stage of disease, treatment regimen, and treatment facility, the general population had better overall survival than Medicaid patients from either the highest-income (HR 1.130, *p* < 0.001; Figure 1d) or highest-education (HR 1.209, *p* < 0.001; Figure 1e) neighborhoods.

## 4. Discussion

The association between socioeconomic inequality and worse patient outcomes is well established and significant but not well understood. Further investigation into multifactorial predictors may reveal interactions that surpass their individual effects on patient experience and thereby offer insight into how these inequalities can be mitigated. To better evaluate this in patients with CRC, we used the NCDB to stratify low-SES patients by the median income and median educational level of their zip codes of residence. This was the first study to evaluate the influence of neighborhood on patients with low individual SES in CRC.

We found that the time from diagnosis to initiation of any treatment was significantly shorter in patients from higher-income or higher-education neighborhoods compared to their peers from low-income or low-education areas. This can be explained for a variety of reasons. Previous research showed that patients living in higher-income areas were more likely to be referred to cancer specialists who worked as part of a multi-disciplinary team [38]. Patients benefit from increased guideline-concordant care and the comprehensive services offered at large academic centers. These centers often have established systems in place to coordinate care and streamline the process for patients, enhancing the overall treatment experience and improving efficiency. The environmental impact on time to initiation of treatment could also be secondary to improved health literacy in the patient’s community. The patient’s support network may prompt them to seek care at major medical centers rather than smaller community hospitals or encourage patients to more readily participate in preventative health (e.g., annual appointments, screening).

The literature has shown that screening tests, like colonoscopies, are less often utilized by individuals with low individual SES and neighborhood SES given competing financial demands on the patients’ time, less access to health care, and high cost of colonoscopy [39,40,41]. However, living in a more affluent neighborhood may provide easier access to these screening tests, whether that be via improved transportation options, greater work flexibility, or other factors. In line with this, we found that patients in high-income or high-education neighborhoods were diagnosed at earlier stages than patients in low-income or low-education neighborhoods. Finally, lifestyle factors, like diet, exercise, tobacco, and/or alcohol use impact our overall health and risk of CRC [42,43,44,45]. Socioeconomic inequality plays a role in these exposures through access to healthy food, exercise options, and health education. For individuals with low SES, living in a community that enables healthy choices may impact these modifiable risk factors.

It is well established that patients with CRC living in lower-income areas experience worse outcomes [16,17,28,29,30,31,32,33]. Our study took this one step further by evaluating a group of patients with low individual SES to distinguish the role of the environment from the individual’s SES. We found that living in a higher-income or higher-education area was associated with improved overall survival. This also held true when comparing survival in the HI/HE and LI/LE cohorts. The improvement in survival may be attributed to a shorter time between diagnosis and treatment, improved lifestyle choices, or increased access to screening and earlier stage at diagnosis. However, when comparing Medicaid patients in the high-income or high-education areas to the general population, Medicaid patients still had worse overall survival. This suggests that while environmental factors can be leveraged to improve outcomes, a patient’s individual low SES cannot be entirely mitigated by their surroundings.

Our study is inherently limited by its retrospective nature. We chose the NCDB as our primary data source because it captures 75% of the newly diagnosed cancer cases in the United States and had the patient demographics, tumor characteristics, and treatment details necessary for this study. However, future studies could incorporate additional data sources (e.g., census data) for more granular socioeconomic data. For example, this study uses Medicaid as a proxy for low-SES, rather than individualized patient data. Although Medicaid status has been used in this way before [36], it has its limitations since some patients with increased medical needs qualify for Medicaid without meeting the income-based threshold (e.g., end-stage renal disease on dialysis). This can potentially confound some of the results that we observed. Additionally, Medicaid eligibility varies geographically, so this population is not necessarily uniform in its SES, especially when compared to non-Medicaid patients. Finally, the NCDB only provides primary payor information, so we were unable to separate Medicaid recipients from dually Medicaid-/Medicare-insured patients. Future investigation into this question would ideally use individual-level income and education data to avoid this limitation.

Additionally, this study was not able to control for individual education level. Low-income patients may individually have high levels of education and derive direct benefit from such, rather than getting an indirect benefit through their surroundings. The time from diagnosis to surgery variable is limited in its interpretation due to evolving practice changes over the past two decades in CRC and the different treatment pathways for colon versus rectal cancer. Finally, our combined variable analysis was limited given the small number of patients living in the lowest-income/highest-education zip codes (n = 79) and highest-income/lowest-education zip codes (n = 1317). Given these low numbers, we were unable to draw robust conclusions from this analysis on the interaction between the two variables.

## 5. Conclusions

In conclusion, Medicaid patients derive an environmental benefit from their surroundings when they live in more affluent neighborhoods. This association is likely driven by faster treatment initiation in higher-SES neighborhoods. However, despite the improvement in survival, these patients still lag behind their non-Medicaid counterparts. Future studies should focus on understanding the mechanisms by which neighborhood SES relates to cancer outcomes to inform targeted interventions and help close the survival gap related to socioeconomic inequality.

## Figures and Tables

**Table 1:** Univariable analysis for variables by income. Baseline comparison between Medicaid patients diagnosed with colorectal cancer living in zip codes with the highest-quartile median income vs. those in living in the zip codes with the lowest-quartile median income. Values in parentheses represent percentages or standard deviation (^†^).

	Low Income	High Income	*p*-Value
	15,730 (54.7%)	13,046 (45.3%)	
Age at Diagnosis ^†^	55.615 (11.974)	57.660 (14.012)	<0.001
Sex			
Male	7873 (50.1%)	6428 (49.3%)	0.188
Female	7857 (49.9%)	6618 (50.7%)	
Race			
White	8046 (51.2%)	8884 (68.1%)	<0.001
Black	6607 (42.0%)	1940 (14.9%)	
Asian	422 (2.7%)	1508 (11.6%)	
Other	655 (4.2%)	714 (5.5%)	
Ethnicity			
Non-Hispanic	12,716 (83.7%)	11,031 (87.4%)	<0.001
Hispanic	2485 (16.3%)	1584 (12.6%)	
Charlson-Deyo Score ^†^	0.454 (0.800)	0.363 (0.725)	<0.001
Facility Type			
Community Cancer Program	1262 (8.8%)	1155 (9.8%)	<0.001
Comprehensive Community Cancer Program	4403 (30.8%)	4010 (34.0%)	
Academic/Research Program	6292 (44.0%)	4321 (36.6%)	
Integrated Network Cancer Program	2343 (16.4%)	2315 (19.6%)	
Clinical Stage			
0	653 (8.7%)	502 (8.2%)	0.001
1	1224 (16.2%)	1139 (18.7%)	
2	1003 (13.3%)	836 (13.7%)	
3	808 (10.7%)	675 (11.1%)	
4	3851 (51.1%)	2948 (48.3%)	
Received Chemotherapy			
No	7992 (50.8%)	6838 (52.4%)	0.023
Yes	7199 (45.8%)	5764 (44.2%)	
Unknown	539 (3.4%)	444 (3.4%)	
Received Immunotherapy			
No	14,589 (92.8%)	12,277 (94.1%)	<0.001
Yes	1016 (6.5%)	664 (5.1%)	
Unknown	124 (0.8%)	105 (0.8%)	
Received Surgery			
No	3019 (19.3%)	2304 (17.7%)	<0.001
Yes	12,661 (80.7%)	10,715 (82.3%)	

**Table 2:** Adjusted outcomes for income. Comparison between Medicaid patients diagnosed with colorectal cancer living in zip codes with the highest-quartile median income vs. those in living in the zip codes with the lowest-quartile median income.

	Estimate (Ref: Low-Income)	95% Confidence Interval	*p*-Value
Time to treatment initiation ^†^	−1.847	−3.342, −0.353	0.015
Time to surgery ^†^	−1.390	−4.014, 1.233	0.299
30-day postoperative mortality ^‡^	0.730	0.573, 1.098	0.163
90-day postoperative mortality ^‡^	0.863	0.684, 1.088	0.211
Overall survival ^§^	0.810	0.768, 0.855	<0.001

All outcomes adjusted for age, sex, race, ethnicity, Charlson–Deyo score, facility type, and disease stage. Postoperative mortality additionally adjusted for presence/absence of chemotherapy and immunotherapy. Overall survival additionally adjusted for presence/absence of chemotherapy, immunotherapy, and surgery. ^†^ Multivariable linear regression; estimate represents beta coefficient. ^‡^ Multivariable logistic regression; estimate represents odds ratio. ^§^ Multivariable Cox regression; estimate represents hazard ratio.

**Table 3:** Univariable analysis for variables by education. Baseline comparison between Medicaid patients diagnosed with colorectal cancer living in zip codes with the highest-quartile high school graduation rate vs. those in living in the zip codes with the lowest-quartile high school graduation rate. Values in parentheses represent percentages or standard deviation (^†^).

	Less Education	More Education	*p*-Value
	19,073 (69.5%)	8375 (30.5%)	
Age at Diagnosis ^†^	55.613 (12.517)	57.500 (13.976)	<0.001
Sex			
Male	9510 (49.9%)	4156 (49.6%)	0.717
Female	9563 (50.1%)	4219 (50.4%)	
Race			
White	10,577 (55.5%)	6173 (73.7%)	<0.001
Black	6234 (32.7%)	1115 (13.3%)	
Asian	1270 (6.7%)	718 (8.6%)	
Other	992 (5.2%)	369 (4.4%)	
Ethnicity			
Non-Hispanic	13,730 (74.0%)	7466 (93.1%)	<0.001
Hispanic	4831 (26.0%)	552 (6.9%)	
Charlson-Deyo Score ^†^	0.404 (0.761)	0.367 (0.728)	<0.001
Facility Type			
Community Cancer Program	1737 (10.1%)	646 (8.5%)	<0.001
Comprehensive Community Cancer Program	5203 (30.2%)	2711 (35.8%)	
Academic/Research Program	7801 (45.3%)	2455 (32.4%)	
Integrated Network Cancer Program	2485 (14.4%)	1758 (23.2%)	
Clinical Stage			
0	767 (8.5%)	285 (7.3%)	0.002
1	1466 (16.3%)	724 (18.6%)	
2	1253 (13.9%)	553 (14.2%)	
3	958 (10.6%)	440 (11.3%)	
4	4559 (50.6%)	1893 (48.6%)	
Received Chemotherapy			
No	9697 (50.8%)	4315 (51.5%)	0.009
Yes	8669 (45.5%)	3811 (45.5%)	
Unknown	707 (3.7%)	249 (3.0%)	
Received Immunotherapy			
No	17,793 (93.3%)	7900 (94.3%)	0.005
Yes	1103 (5.8%)	411 (4.9%)	
Unknown	176 (0.9%)	64 (0.8%)	
Received Surgery			
No	3607 (19.0%)	1442 (17.3%)	<0.001
Yes	15,415 (81.0%)	6914 (82.7%)	

**Table 4:** Adjusted outcomes for education. Comparison between Medicaid patients diagnosed with colorectal cancer living in zip codes with the highest-quartile high school graduation rate vs. those in living in the zip codes with the lowest-quartile high school graduation rate.

	Estimate (Ref: Low-Education)	95% Confidence Interval	*p*-Value
Time to treatment initiation ^†^	−3.926	−5.643, −2.210	<0.001
Time to surgery ^†^	−2.500	−5.456, 0.457	0.097
30-day postoperative mortality ^‡^	1.047	0.734, 1.494	0.799
90-day postoperative mortality ^‡^	0.950	0.737, 1.225	0.694
Overall survival ^§^	0.897	0.845, 0.951	<0.001

All outcomes adjusted for age, sex, race, ethnicity, Charlson–Deyo score, facility type, and disease stage. Postoperative mortality additionally adjusted for presence/absence of chemotherapy and immunotherapy. Overall survival additionally adjusted for presence/absence of chemotherapy, immunotherapy, and surgery. ^†^ Multivariable linear regression; estimate represents beta coefficient. ^‡^ Multivariable logistic regression; estimate represents odds ratio. ^§^ Multivariable Cox regression; estimate represents hazard ratio.

**Table 5:** Adjusted outcomes for combination income/education analysis.

	Comparison Group	Estimate (Ref: LI/LE)	95% Confidence Interval	*p*-Value
Time to treatment initiation ^†^	LI/HE	−7.642	−16.218, 0.933	0.081
	HI/LE	−1.465	−6.376, 3.446	0.559
	HI/HE	−4.419	−6.485, −2.353	<0.001
Time to surgery ^†^	LI/HE	−4.816	−19.990, 10.359	0.534
	HI/LE	−7.458	−16.543, 1.626	0.108
	HI/HE	−3.613	−7.449, 0.224	0.065
30-day postoperative mortality ^‡^	LI/HE	0.905	0.196, 4.183	0.898
	HI/LE	0.963	0.277, 3.344	0.953
	HI/HE	1.057	0.677, 1.651	0.807
90-day postoperative mortality ^‡^	LI/HE	0.772	0.244, 2.437	0.659
	HI/LE	0.753	0.307, 1.848	0.307
	HI/HE	1.039	0.760, 1.422	0.809
Overall survival ^§^	LI/HE	0.958	0.717, 1.278	0.768
	HI/LE	0.800	0.661, 0.968	0.022
	HI/HE	0.827	0.768, 0.890	<0.001

LI/LE: low-income, low-education; LI/HE: low-income, high-education; HI/LE: high-income, low-education; HI/HE: high-income, high-education. All outcomes adjusted for age, sex, race, ethnicity, Charlson–Deyo score, facility type, and disease stage. Postoperative mortality additionally adjusted for presence/absence of chemotherapy and immunotherapy. Overall survival additionally adjusted for presence/absence of chemotherapy, immunotherapy, and surgery. ^†^ Multivariable linear regression; estimate represents beta coefficient. ^‡^ Multivariable logistic regression; estimate represents odds ratio. ^§^ Multivariable Cox regression; estimate represents hazard ratio.

**Table 6:** Adjusted overall survival for high-SES Medicaid vs. non-Medicaid comparison.

Comparison Group	Hazard Ratio (Ref: Non-Medicaid)	95% Confidence Interval	*p*-Value
High-income Medicaid	1.130	1.088, 1.173	<0.001
High-education Medicaid	1.209	1.155, 1.265	<0.001

Multivariable Cox regression. Outcomes adjusted for age, sex, race, ethnicity, Charlson–Deyo score, facility type, and disease stage, and presence/absence of chemotherapy, immunotherapy, and surgery.

## Data Availability

The data presented in this study are available in the National Cancer Database (NCDB) at https://www.facs.org/quality-programs/cancer-programs/national-cancer-database/ (accessed on 16 April 2025) [34].

## References

[1] Siegel R.L., Giaquinto A.N., Jemal A. (2024). Cancer statistics, 2024. CA A Cancer J. Clin..

[2] Morgan E., Arnold M., Gini A., Lorenzoni V., Cabasag C.J., Laversanne M., Vignat J., Ferlay J., Murphy N., Bray F. (2023). Global burden of colorectal cancer in 2020 and 2040: Incidence and mortality estimates from GLOBOCAN. Gut.

[3] Issaka R.B., Chan A.T., Gupta S. (2023). AGA Clinical Practice Update on Risk Stratification for Colorectal Cancer Screening and Post-Polypectomy Surveillance: Expert Review. Gastroenterology.

[4] Benson A.B., Venook A.P., Adam M., Chang G., Chen Y.-J., Ciombor K.K., Cohen S.A., Cooper H.S., Deming D., Garrido-Laguna I. (2024). Colon Cancer, Version 3.2024, NCCN Clinical Practice Guidelines in Oncology. J. Natl. Compr. Cancer Netw..

[5] Benson A.B., Venook A.P., Adam M., Chang G., Chen Y.-J., Ciombor K.K., Cohen S.A., Cooper H.S., Deming D., Garrido-Laguna I. (2024). NCCN Guidelines® Insights: Rectal Cancer, Version 3.2024. J. Natl. Compr. Cancer Netw..

[6] Turrell G., Kavanagh A., Draper G., Subramanian S.V. (2007). Do places affect the probability of death in Australia? A multilevel study of area-level disadvantage, individual-level socioeconomic position and all-cause mortality, 1998–2000. J. Epidemiol. Community Health.

[7] Li X., Sundquist J., Zöller B., Sundquist K. (2015). Neighborhood Deprivation and Lung Cancer Incidence and Mortality: A Multilevel Analysis from Sweden. J. Thorac. Oncol..

[8] Goel N., Hernandez A., Thompson C., Choi S., Westrick A., Stoler J., Antoni M.H., Rojas K., Kesmodel S., Figueroa M.E. (2023). Neighborhood Disadvantage and Breast Cancer–Specific Survival. JAMA Netw. Open.

[9] Bentley R., Kavanagh A.M., Subramanian S.V., Turrell G. (2007). Area disadvantage, individual socio-economic position, and premature cancer mortality in Australia 1998 to 2000: A multilevel analysis. Cancer Causes Control.

[10] van Loon A., Brandt P.v.D., Golbohm R. (1995). Socioeconomic status and colon cancer incidence: A prospective cohort study. Br. J. Cancer.

[11] Kim D., Masyn K.E., Kawachi I., Laden F., Colditz G.A. (2010). Neighborhood socioeconomic status and behavioral pathways to risks of colon and rectal cancer in women. Cancer.

[12] Doubeni C.A., Laiyemo A.O., Major J.M., Schootman M., Lian M., Park Y., Graubard B.I., Hollenbeck A.R., Sinha R. (2012). Socioeconomic status and the risk of colorectal cancer. Cancer.

[13] Zhang D., Matthews C.E., Powell-Wiley T.M., Xiao Q. (2018). Ten-year change in neighborhood socioeconomic status and colorectal cancer. Cancer.

[14] Schwartz K.L., Crossley-May H., Vigneau F.D., Brown K., Banerjee M. (2003). Race, socioeconomic status and stage at diagnosis for five common malignancies. Cancer Causes Control.

[15] Frederiksen B.L., Osler M., Harling H., Jørgensen T., On behalf of Danish Colorectal Cancer Group (2008). Social inequalities in stage at diagnosis of rectal but not in colonic cancer: A nationwide study. Br. J. Cancer.

[16] Salem M.E., Puccini A., Trufan S.J., Sha W., Kadakia K.C., Hartley M.L., Musselwhite L.W., Symanowski J.T., Hwang J.J., Raghavan D. (2021). Impact of Sociodemographic Disparities and Insurance Status on Survival of Patients with Early-Onset Colorectal Cancer. Oncologist.

[17] Ko T.M., Laraia K.N., Alexander H.R., Ecker B.L., Grandhi M.S., Kennedy T.J., In H., Langan R.C., Pitt H.A., Stroup A.M. (2024). Low neighborhood socioeconomic status is associated with poor outcomes in young adults with colorectal cancer. Surgery.

[18] Yousef M., Yousef A., Chowdhury S., Fanaeian M.M., Knafl M., Peterson J., Zeineddine M., Alfaro K., Zeineddine F., Goldstein D. (2024). Molecular, Socioeconomic, and Clinical Factors Affecting Racial and Ethnic Disparities in Colorectal Cancer Survival. JAMA Oncol..

[19] Jansen L., Eberle A., Emrich K., Gondos A., Holleczek B., Kajüter H., Maier W., Nennecke A., Pritzkuleit R., Brenner H. (2013). Socioeconomic deprivation and cancer survival in Germany: An ecological analysis in 200 districts in Germany. Int. J. Cancer.

[20] Tham N.L., Skandarajah A., Hayes I.P. (2022). Socioeconomic disadvantage and its impact on colorectal cancer in Australia: A scoping review. ANZ J. Surg..

[21] Blair A., Datta G.D. (2020). Associations between area-level deprivation, rural residence, physician density, screening policy and late-stage colorectal cancer in Canada. Cancer Epidemiol..

[22] Renna Junior N.L., Silva G.d.A.e. (2023). Socioeconomic status and cancer survival in Brazil: Analysis of population data from the municipalities of Aracaju and Curitiba, 1996–2012. Cancer Epidemiol..

[23] Kaneko N., Nishino Y., Ito Y., Nakaya T., Kanemura S. (2023). Association of Socioeconomic Status Assessed by Areal Deprivation With Cancer Incidence and Detection by Screening in Miyagi, Japan Between 2005 and 2010. J. Epidemiol..

[24] Lee J., Park J., Kim N., Nari F., Bae S., Lee H.J., Lee M., Jun J.K., Choi K.S., Suh M. (2024). Socioeconomic Disparities in Six Common Cancer Survival Rates in South Korea: Population-Wide Retrospective Cohort Study. JMIR Public Health Surveill..

[25] Shaw C., Blakely T., Sarfati D., Fawcett J., Peace J. (2006). Trends in colorectal cancer mortality by ethnicity and socio-economic position in New Zealand, 1981–99: One country, many stories. Aust. N. Z. J. Public Health.

[26] Egeberg R., Halkjær J., Rottmann N., Hansen L., Holten I. (2008). Social inequality and incidence of and survival from cancers of the colon and rectum in a population-based study in Denmark, 1994–2003. Eur. J. Cancer.

[27] GlobalSurg C. (2021). National Institute for Health Research Global Health Research Unit on Global S. Global variation in postoperative mortality and complications after cancer surgery: A multicentre, prospective cohort study in 82 countries. Lancet.

[28] Du X.L., Fang S., Vernon S.W., El-Serag H., Shih Y.T., Davila J., Rasmus M.L. (2007). Racial disparities and socioeconomic status in association with survival in a large population-based cohort of elderly patients with colon cancer. Cancer.

[29] Niu X., Pawlish K.S., Roche L.M. (2010). Cancer Survival Disparities by Race/Ethnicity and Socioeconomic Status in New Jersey. J. Health Care Poor Underserved.

[30] Hines R., Markossian T., Johnson A., Dong F., Bayakly R. (2014). Geographic Residency Status and Census Tract Socioeconomic Status as Determinants of Colorectal Cancer Outcomes. Am. J. Public Health.

[31] Tannenbaum S.L., Hernandez M., Zheng D.D., Sussman D.A., Lee D.J. (2014). Individual- and Neighborhood-Level Predictors of Mortality in Florida Colorectal Cancer Patients. PLoS ONE.

[32] A Hastert T., A A Beresford S., Sheppard L., White E. (2014). Disparities in cancer incidence and mortality by area-level socioeconomic status: A multilevel analysis. J. Epidemiol. Community Health.

[33] Zhu B., Hu F.-H., Jia Y.-J., Zhao D.-Y., Zhang W.-Q., Tang W., Hu S.-Q., Ge M.-W., Du W., Shen W.-Q. (2023). Socioeconomic status on survival outcomes in patients with colorectal cancer: A cross-sectional study. J. Cancer Res. Clin. Oncol..

[34] Habermann E.B., Day C.N., EPalis B., Plichta J.K.M.F., Wasif N.M., Weigel R.J.M., Boughey J.C.M. (2024). American College of Surgeons Cancer Program Annual Report from 2021 Participant User File. J. Am. Coll. Surg..

[35] Donohue J.M., Cole E.S., James C.V., Jarlenski M., Michener J.D., Roberts E.T. (2022). The US Medicaid Program. JAMA.

[36] Lewis V.A., Maddox K.J., Austin A.M., Gottlieb D.J., Bynum J.P. (2019). Developing and Validating a Measure to Estimate Poverty in Medicare Administrative Data. Med. Care.

[37] Deyo R.A., Cherkin D.C., Ciol M.A. (1992). Adapting a clinical comorbidity index for use with ICD-9-CM administrative databases. J. Clin. Epidemiol..

[38] Goulart B.H., Reyes C.M., Fedorenko C.R., Mummy D.G., Satram-Hoang S., Koepl L.M., Blough D.K., Ramsey S.D. (2013). Referral and Treatment Patterns Among Patients with Stages III and IV Non–Small-Cell Lung Cancer. J. Oncol. Pract..

[39] Doubeni C.A., Laiyemo A.O., Reed G., Field T.S., Fletcher R.H. (2009). Socioeconomic and Racial Patterns of Colorectal Cancer Screening among Medicare Enrollees in 2000 to 2005. Cancer Epidemiol. Biomark. Prev..

[40] Lian M., Schootman M., Yun S. (2008). Geographic variation and effect of area-level poverty rate on colorectal cancer screening. BMC Public Health.

[41] Khan M.M.M., Munir M.M., Woldesenbet S., Endo Y., Khalil M., Tsilimigras D., Harzman A., Huang E., Kalady M., Pawlik T.M. (2024). Association of COVID-19 Pandemic with Colorectal Cancer Screening: Impact of Race/Ethnicity and Social Vulnerability. Ann. Surg. Oncol..

[42] Platz E.A., Willett W.C., Colditz G.A., Rimm E.B., Spiegelman D., Giovannucci E. (2000). Proportion of colon cancer risk that might be preventable in a cohort of middle-aged US men. Cancer Causes Control.

[43] Aleksandrova K., Pischon T., Jenab M., Bueno-De-Mesquita H.B., Fedirko V., Norat T., Romaguera D., Knüppel S., Boutron-Ruault M.-C., Dossus L. (2014). Combined impact of healthy lifestyle factors on colorectal cancer: A large European cohort study. BMC Med..

[44] Botteri E., Peveri G., Berstad P., Bagnardi V., Chen S.L., Sandanger T.M., Hoff G., Dahm C.C., Antoniussen C.S., Tjønneland A. (2022). Changes in Lifestyle and Risk of Colorectal Cancer in the European Prospective Investigation into Cancer and Nutrition. Am. J. Gastroenterol..

[45] Torres Stone R.A., EWaring M., Cutrona S.L., IKiefe C., Allison J., ADoubeni C. (2017). The association of dietary quality with colorectal cancer among normal weight, overweight and obese men and women: A prospective longitudinal study in the USA. BMJ Open.

